# IntSplice2: Prediction of the Splicing Effects of Intronic Single-Nucleotide Variants Using LightGBM Modeling

**DOI:** 10.3389/fgene.2021.701076

**Published:** 2021-07-19

**Authors:** Jun-ichi Takeda, Sae Fukami, Akira Tamura, Akihide Shibata, Kinji Ohno

**Affiliations:** ^1^Division of Neurogenetics, Center for Neurological Diseases and Cancer, Nagoya University Graduate School of Medicine, Nagoya, Japan; ^2^Department of Anesthesiology, Toranomon Hospital, Tokyo, Japan

**Keywords:** splice acceptor site, aberrant splicing, single nucleotide variations, intronic mutations, LightGBM

## Abstract

Prediction of the effect of a single-nucleotide variant (SNV) in an intronic region on aberrant pre-mRNA splicing is challenging except for an SNV affecting the canonical GU/AG splice sites (ss). To predict pathogenicity of SNVs at intronic positions −50 (Int-50) to −3 (Int-3) close to the 3’ ss, we developed light gradient boosting machine (LightGBM)-based IntSplice2 models using pathogenic SNVs in the human gene mutation database (HGMD) and ClinVar and common SNVs in dbSNP with 0.01 ≤ minor allelic frequency (MAF) < 0.50. The LightGBM models were generated using features representing splicing *cis*-elements. The average recall/sensitivity and specificity of IntSplice2 by fivefold cross-validation (CV) of the training dataset were 0.764 and 0.884, respectively. The recall/sensitivity of IntSplice2 was lower than the average recall/sensitivity of 0.800 of IntSplice that we previously made with support vector machine (SVM) modeling for the same intronic positions. In contrast, the specificity of IntSplice2 was higher than the average specificity of 0.849 of IntSplice. For benchmarking (BM) of IntSplice2 with IntSplice, we made a test dataset that was not used to train IntSplice. After excluding the test dataset from the training dataset, we generated IntSplice2-BM and compared it with IntSplice using the test dataset. IntSplice2-BM was superior to IntSplice in all of the seven statistical measures of accuracy, precision, recall/sensitivity, specificity, F1 score, negative predictive value (NPV), and matthews correlation coefficient (MCC). We made the IntSplice2 web service at https://www.med.nagoya-u.ac.jp/neurogenetics/IntSplice2.

## Introduction

RNA splicing is an essential process to generate mature mRNAs from precursor mRNAs, especially in higher eukaryotes (Crick, 1979). RNA splicing is performed by a spliceosome complex, the major constituents of which are five small nuclear ribonucleoproteins (snRNPs) named U1, U2, U4, U5, and U6 (Wahl et al., 2009). In the spliceosomal E complex at the first stage of splicing, U1 snRNP binds to the 5’ splice sites (ss) spanning the “GU” dinucleotide; SF1 binds to the branch point sequence (BPS); U2AF65 binds to the polypyrimidine tract (PPT); U2AF35 binds to the intron/exon boundary spanning the “AG” dinucleotide; and accessory splicing factors like serine–arginine-rich splicing factors (SRSFs) and heterologous nuclear ribonucleoproteins (hnRNPs) bind to their cognate exonic/intronic sequences (Ohno et al., 2018). Spatiotemporal regulation of the accessory splicing factors enables tissue-specific and developmental stage-specific regulation of alternative splicing events that are observed in 92–94% of human multi-exon genes (Wang et al., 2008). Constitutive and alternative splicing events are sometimes affected by single-nucleotide variants (SNVs) located not only at “GU/AG” dinucleotides but also at deep introns or even exons. A plethora of tools have been reported to predict exonic SNVs that cause aberrant splicing (Cartegni et al., 2003; Fairbrother et al., 2004; Wang et al., 2004; Zhang and Chasin, 2004; Zhang et al., 2005; Goren et al., 2006; Desmet et al., 2009; Divina et al., 2009; Piva et al., 2009, 2012; Paz et al., 2010; Lim et al., 2011; Chang et al., 2013). We previously developed a support vector machine (SVM)-based model, IntSplice, that predicts the effects on splicing of intronic SNVs (Int-SNVs) at positions from intronic position −50 (Int-50) to Int-3 (Shibata et al., 2016). The gradient boosting (GB) modeling produces competitive, highly robust, and interpretable procedures for both regression and classification (Friedman, 2001). In this study, we developed IntSplice2 using newly available SNV datasets and light gradient boosting machine (LightGBM) (Ke et al., 2017), which is a free and open-source distributed GB framework that uses tree-based learning algorithms.

## Materials and Methods

### Annotated SNVs to Generate IntSplice2

The major pipelines of our analysis are indicated in Supplementary Figure 1. We used the human gene mutation database (HGMD) professional release April 2020 (Stenson et al., 2017) with mutation category DM (disease-causing mutation) and ClinVar release March 15, 2021 (Landrum et al., 2018) with CLNVC = single_nucleotide_variant and intron_variant, and CLNSIG = pathogenic to obtain pathogenic SNVs on the human genome assembly GRCh38/hg38 (NCBI Resource Coordinators, 2018). We extracted 1,787 pathogenic SNVs located from Int-50 to Int-3 preceding internal coding exons according to the transcript annotations of Ensembl release 101 (Howe et al., 2021). We then randomly extracted 1,787 common SNVs out of 5,406 common SNVs with a minor allelic frequency (MAF) between 0.01 and 0.50 at positions from Int-50 to Int-3 preceding internal coding exons from dbSNP build 151 (Sherry et al., 2001) on GRCh38/hg38 with VC = SNV (Annotated Dataset-1787 in Supplementary Figure 1). To compare common SNVs with MAFs < 0.50 and < 0.99 in generating IntSplice2 models, we randomly extracted 1,787 common SNVs out of 33,252 common SNVs with 0.01 ≤ MAF < 0.99.

### Features to Generate IntSplice2

To make IntSplice2 models, we used essentially the same 110 features that were used to make IntSplice (Shibata et al., 2016). Briefly, these features included exon length, the number of pyrimidines in the PPT, the position of predicted BPS, the sequence of predicted BPS, individual nucleotides at intron −3 and exon +1, the strength of splicing signals at the 5 and 3’ ss, and motifs of RNA-binding proteins predicted by SpliceAid 2 (Piva et al., 2012), to name a few (Supplementary Table 1). We added these features to Annotated Dataset-1787 to make Training Dataset-1787 (Supplementary Figure 1).

### Generation and Evaluation of IntSplice2

To make IntSplice2 models using Training Dataset-1787, we first optimized hyperparameters with Optuna (Akiba et al., 2019) and then used LightGBM (Ke et al., 2017) with the optimized hyperparameters on Python version 3.8. The hyperparameters used to make an IntSplice2 model are shown in Supplementary Table 2. We evaluated the performance of IntSplice2 models by fivefold cross-validation (CV) with the area under the receiver operating characteristic curve (AUROC) and the area under the precision/recall curve (AUPR), as well as with seven statistical measures composed of accuracy, precision, recall/sensitivity, specificity, F1 score, negative predictive value (NPV), and matthews correlation coefficient (MCC), which were recommended in the Human Mutation guidelines (Vihinen, 2013; Grimm et al., 2015).

### Generation of IntSplice2-BM to Be Compared With IntSplice

For benchmarking (BM) the performance of LightGBM-based IntSplice2 against that of SVM-based IntSplice, we made Test Dataset-288 that was composed of 288 pathogenic and 288 common SNVs with 0.01 ≤ MAF < 0.50, which were not included in the Training Dataset for IntSplice (Supplementary Figure 1). Exclusion of Test Dataset-288 from Training Dataset-1787 generated Training Dataset-1499. We made IntSplice2-BM using Training Dataset-1499. Thus, Test Dataset-288 had no circularity with either IntSplice2-BM or IntSplice. IntSplice2-BM and IntSplice were evaluated by the seven statistical measures in the Human Mutation guidelines (Vihinen, 2013; Grimm et al., 2015). As SVM-based IntSplice was a binary classifier with a fixed threshold, AUROC, and AUPR could not be calculated for IntSplice.

## Results

### IntSplice2 Models Generated by LightGBM Modeling

**FIGURE 1:**
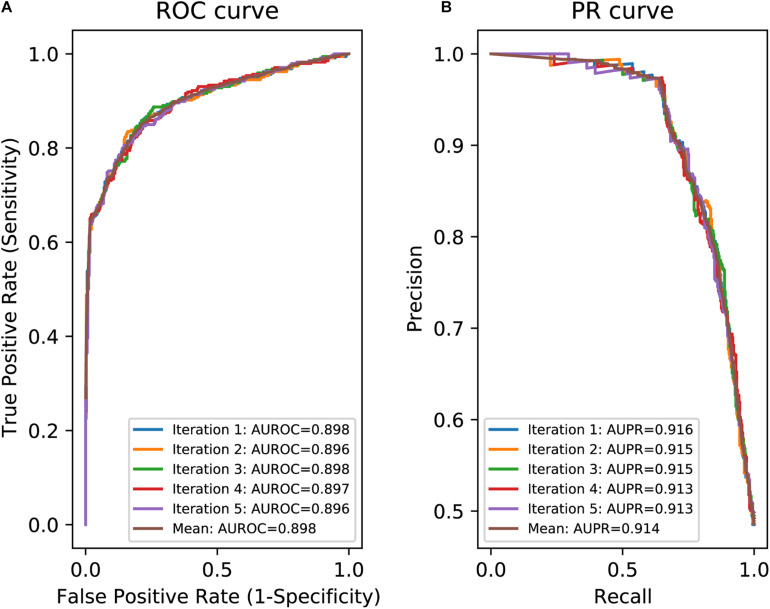
Evaluation of IntSplice2 by fivefold CV. **(A)** Five iterated and mean ROC curves with AUROCs. **(B)** Five iterated and mean PR curves with AUPRs.

**FIGURE 2:**
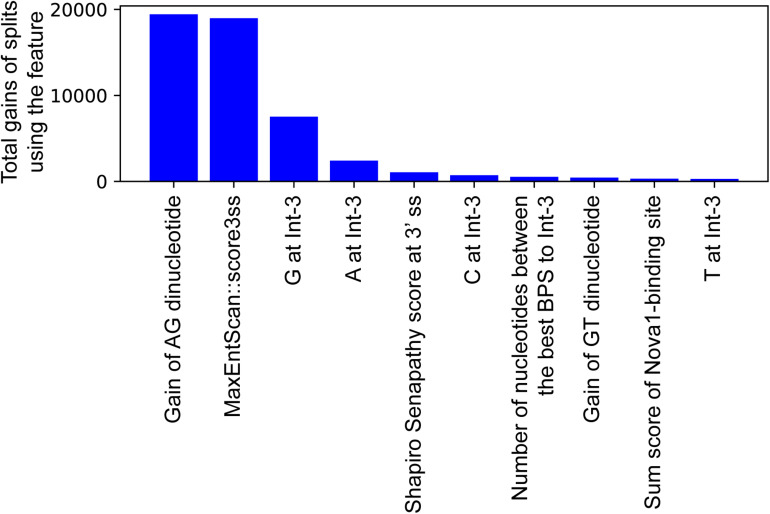
The top 10 important features of IntSplice2 in 110 features.

In an effort to make a new dependable model to predict the splicing effects of Int-SNVs at positions from Int-50 to Int-3, we made IntSplice2 using newly available SNVs and LightGBM modeling. We first asked whether the inclusion or exclusion of major SNVs that are observed in more than 50% in humans would improve the performance of generated models. We compared IntSplice2 models generated using common SNVs with 0.01 ≤ MAF < 0.50 and with 0.01 ≤ MAF < 0.99 by fivefold CV. SNVs with MAF > 0.50 indicate that the reference nucleotide represents a minor nucleotide. We found that common SNVs with 0.01 ≤ MAF < 0.50 gave rise to better scores in seven out of nine statistical measures than those with 0.01 ≤ MAF < 0.99 (Supplementary Table 3). We thus chose 0.01 ≤ MAF < 0.50 to generate IntSplice2 (Training Dataset-1787). The seven statistical measures (accuracy, precision, recall/sensitivity, specificity, F1 score, NPV, and MCC) of IntSplice2 by fivefold CV of the Training Dataset-1787 are shown in Table 1. IntSplice2 exhibited an average recall/sensitivity [true-positive rate (TPR)] of 0.764 and an average specificity of 0.884. We previously reported that IntSplice had an average recall/sensitivity of 0.800 and an average specificity of 0.849 (Shibata et al., 2016). Thus, IntSplice2 had a lower false-positive rate (FPR) at the cost of a higher false-negative rate (FNR) compared to IntSplice. The receiver operating characteristic (ROC) and precision/recall (PR) curves of IntSplice2 by fivefold CV are shown in Figure 1. The average AUROC and AUPR were 0.898 and 0.914, respectively. ROC and PR curves of IntSplice could not be drawn because IntSplice was a binary classifier with a fixed threshold (Shibata et al., 2016). The best feature importance of IntSplice2 was “Gain of AG dinucleotide” (Figure 2). Similarly, the following features were ranked from second to fifth: “MaxEntScan::score3ss” (Yeo and Burge, 2004), “G at Int-3,” “A at Int-3,” and “Shapiro Senapathy score at the 3’ ss,” respectively. We previously reported that “G at Int-3” is frequently observed in exons that are alternatively skipped in the human genome (Shibata et al., 2016). In addition, both cryo-electron microscopy and isothermal titration calorimetry show that “G at Int-3” decreases a binding affinity for U2AF35 (Yoshida et al., 2020).

**TABLE 1:** Seven statistical measures indicated in the Human Mutation guidelines (Vihinen, 2013; Grimm et al., 2015) of IntSplice2 by fivefold CV of Training Dataset-1787.

	**Accuracy**	**Precision**	**Recall/sensitivity**	**Specificity**	**F1 score**	**NPV**	**MCC**
IntSplice2	0.826	0.861	0.764	0.884	0.809	0.800	0.654

Accuracy = $\frac{\text{TP}+\text{TN}}{\text{TP}+\text{FP}+\text{TN}+\text{FN}}$							
Rate to predict true positives and true negatives in the whole dataset							
Precision/Positive Prediciton Value (PPV) = $\frac{\text{TP}}{\text{TP}+\text{FP}}$							
Rate of true positives in predicted positives							
Recall/Sensitivity = $\frac{\text{TP}}{\text{TP}+\text{FN}}$							
Rate of true positives in actual positives							
Specificity = $\frac{\text{TN}}{\text{FP}+\text{TN}}$							
Rate of true negatives in actual negatives							
F1 score = $2\,\frac{\text{Precision}\times \text{Recall}}{\text{Precision}+\text{Recall}}$							
Harmonic mean of precision and recall. Higher precision and higher recall increase F1 score, but discrepancy between precision and recall lowers F1 score							
NPV = $\frac{\text{TN}}{\text{TN}+\text{FN}}$							
Rate of true negatives in predicted negatives							
MCC = $\frac{\text{TP}\times \text{TN}-\text{FP}\times \text{FN}}{\sqrt{\left(\mathrm{TP}+\mathrm{FP}\right)\,\left(\mathrm{TP}+\mathrm{FN}\right)\,\left(\mathrm{TN}+\mathrm{FP}\right)\,\left(\mathrm{TN}+\mathrm{FN}\right)}}$							
A correlation coefficient between the actual and predicted binary conditions while the numbers of each condition are balanced. Unlike the other parameters, MCC balances the ratio between actual positives and actual negatives.							
**Confusion matrix:**							
					**Actual condition**		

					**Actual positive**		**Actual negative**

Predicted condition		Predicted positive			True positive (TP)		False positive (FP)
		Predicted negative			False negative (FN)		True negative (TN)

### Comparison of IntSplice2-BM Model With the IntSplice Model

Training Dataset-1787 was divided into Test Dataset-288, which was not used to train IntSplice, and Training Dataset-1499. We generated the IntSplice2-BM model using Training Dataset-1499. The average recall/sensitivity and the average specificity of IntSplice2-BM by fivefold CV were 0.764 and 0.889, respectively. We next compared the performances of IntSplice2-BM and IntSplice models using Test Dataset-288 and found that all the seven statistical measures were higher with the IntSplice2-BM model than the IntSplice model (Table 2).

**TABLE 2:** Seven statistical measures of IntSplice2-BM and IntSplice models using Test Dataset-288, which has no circularity with the respective training datasets.

	**Accuracy**	**Precision**	**Recall/Sensitivity**	**Specificity**	**F1 score**	**NPV**	**MCC**
IntSplice2-BM	0.826	0.873	0.764	0.889	0.815	0.790	0.658
IntSplice	0.802	0.854	0.729	0.875	0.787	0.764	0.611

*IntSplice2-BM, which is a BM model to be compared with IntSplice, is made by Training Dataset-1499.*

### Comparison of LightGBM With Four Other Machine Learning (ML) Methods

We next compared LightGBM with random forest (RF), SVM, extremely randomized trees (ERT), and multilayer perceptron (MLP). The best hyperparameters of RF, SVM, ERT, and MLP were searched for by fivefold CV grid search, and the models were generated using scikit-learn libraries (Pedregosa et al., 2011) on Python version 3.8. Fivefold CV of five models including IntSplice2 made by Training Dataset-1787 showed that LightGBM was ranked first in six of nine statistical measures (Supplementary Table 4A). Similarly, five additional models including IntSplice2-BM made by Training Dataset-1499 were evaluated by Test Dataset-288. We found that LightGBM was ranked first in seven of nine statistical measures (Supplementary Table 4B). Thus, LightGBM was likely to be the best modeling method for our study.

### IntSplice2 Web Service

**FIGURE 3:**
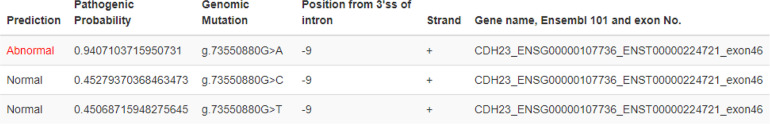
A representative screenshot of the output of IntSplice2 web service. As previously reported, g.73550880G > A on chromosome 10 (GRCh37/hg19) identified in a patient with Usher syndrome is at the ninth nucleotide from the 3’ end of intron 45 of *CDH23*. When a user chooses “GRCh37/hg19” and enters the chromosome number “10” and the genomic coordinate “73550880,” the IntSplice2 web service returns the result on the same window on a browser.

We generated a web service of IntSplice2 at https://www.med.nagoya-u.ac.jp/neurogenetics/IntSplice2. The IntSplice2 web service accepts a genomic coordinate according to either GRCh37/hg19 or GRCh38/hg38. A given coordinate is mapped to all the annotated coding transcripts in Ensembl release 101, and the web service program analyses all the transcripts. The program automatically generates three possible SNVs at the coordinate and predicts a probability of aberrant splicing, where 0 indicates that the SNV should have no effect on splicing and 1 indicates that the SNV should affect splicing of the downstream exon. A probability of aberrant splicing less than 0.5 was predicted to be a splicing-insensitive SNV, whereas that of 0.5 or more was predicted to be a splicing-affecting SNV. When an SNV is located at Int-50 to Int-3 of two or more transcripts, the web service program predicts the effects on splicing for all the relevant transcripts. Representative results are shown in Figure 3. In this example, g.73550880G > A on chromosome 10 (GRCh37/hg19) was predicted to cause aberrant splicing by IntSplice2. This mutation is at intervening sequence (IVS)45−9G > A of *CDH23* and activates a cryptic splice acceptor site with the insertion of seven intronic nucleotides (von Brederlow et al., 2002).

## Discussion

In this study, we generated IntSplice2 using an updated dataset of pathogenic and common SNVs with LightGBM modeling. In contrast to IntSplice2, our previous tool IntSplice used SVM modeling (Shibata et al., 2016). We compared LightGBM-based IntSplice2-BM with SVM-based IntSplice by avoiding circularity between the training and test datasets and found that all the seven statistical measures were better in IntSplice2-BM than in IntSplice (Table 2). We also compared LightGBM-based models with RF-, SVM-, ERT-, and MLP-based models made by two training datasets and found that LightGBM performed the best in most of the nine statistical measures (Supplementary Table 4). Thus, the modeling strategy and the training dataset that we used in IntSplice2 were likely to have enabled us to make a better model compared with IntSplice. The fivefold CV of the training datasets of IntSplice and IntSplice2 showed that the recall/sensitivity of IntSplice2 (0.764) was lower than that of IntSplice (0.800), whereas the specificity of IntSplice2 (0.884) was higher than that of IntSplice (0.849). IntSplice2 was generated using 1,787 pathogenic SNVs, whereas IntSplice was generated using 1,162 pathogenic SNVs. In general, models generated using a larger dataset should be more dependable. In addition, we used LightGBM modeling in IntSplice2. The higher specificity in IntSplice2 was likely to represent that identification of splicing-insensitive nonpathogenic SNVs became more convincing with a larger dataset and with a newer modeling method. The recall/sensitivity, however, was reduced at a cost of increased specificity. The reduced recall/sensitivity may also indicate that features associated with the splicing-affecting SNVs were more diverse than those we predicted with 1,162 pathogenic SNVs in IntSplice.

Recently, four prediction tools were developed using ML approaches for Int-SNVs (Abramowicz and Gos, 2018; Rowlands et al., 2019): RF-based TraP (Gelfman et al., 2017), GB-based S-CAP (Jagadeesh et al., 2019), deep neural network (DNN)-based MMSplice (Cheng et al., 2019), and RF-based RegSNPs-intron (Lin et al., 2019). TraP predicts the effect on splicing of Int-SNVs at any intronic positions, as well as of synonymous exonic SNVs (Gelfman et al., 2017). S-CAP divided an intron–exon–intron region into six subsets of 3′ intronic, 3′ AG core, exonic, 5′ GU core, 5′ extended, and 5′ intronic regions and made a model for each subset (Jagadeesh et al., 2019). The prediction range of the 3′ ss intronic S-CAP model was the same as IntSplice2. RegSNPs-intron predicts the splicing effects of SNVs from Ex-3 to Int+7 for donor sites and from Int-13 to Ex+1 for acceptor sites (Lin et al., 2019). The training datasets and the features used in TraP, S-CAP, and RegSNPs-intron were similar to those in our IntSplice2 and IntSplice models. In these models, the datasets were composed of pathogenic and common SNVs annotated in various databases. Particularly, S-CAP, RegSNPs-intron, IntSplice2, and IntSplice used pathogenic SNVs in the HGMD as annotated data and splicing *cis*-elements as features. RegSNPs-intron additionally used the protein structure and the evolutionary conservation as features. In contrast to these models, MMSplice was a DNN-based model that was trained by true donor and acceptor sites to predict the effects of genetic variants on splicing (Cheng et al., 2019). We compared IntSplice2-BM with TraP, S-CAP, and RegSNPs-intron, whose scores were downloadable, using Test Dataset-288 and found that the statistical measures of IntSplice2-BM were not as good as those of the other three ML tools (Supplementary Table 5). ML tools can be easily overestimated by the presence of circularity, in which a subset of the training dataset is used to evaluate the efficiency of a tool (Grimm et al., 2015; Takeda et al., 2020). In contrast to IntSplice2-BM, TraP, S-CAP, and RegSNPs-intron should have been trained using a substantial number of SNVs in Test Dataset-288, which gave rise to overestimated statistical measures. We hope that the authors of these models will collaborate with each other to make their own models using an identical training dataset for unbiased comparison of the ML models without circularity.

## Data Availability Statement

The original contributions presented in the study are included in the article/Supplementary Material, further inquiries can be directed to the corresponding author.

## Author Contributions

KO conceived the idea. J-iT, SF, AT, and AS designed the methods and performed *in silico* analyses. J-iT, SF, and KO wrote the manuscript. All authors read and approved the final manuscript.

## Conflict of Interest

The authors declare that the research was conducted in the absence of any commercial or financial relationships that could be construed as a potential conflict of interest.

## Supplementary Material

Supplementary Figure 1 The major pipelines of our analysis.
