# Supplementary figures and images for: IntSplice2: Prediction of the Splicing Effects of Intronic Single-Nucleotide Variants Using LightGBM Modeling
Source: Front Genet. 2021 Jul 19;12:701076. doi: 10.3389/fgene.2021.701076 (PMC8326971; doi:10.3389/fgene.2021.701076)

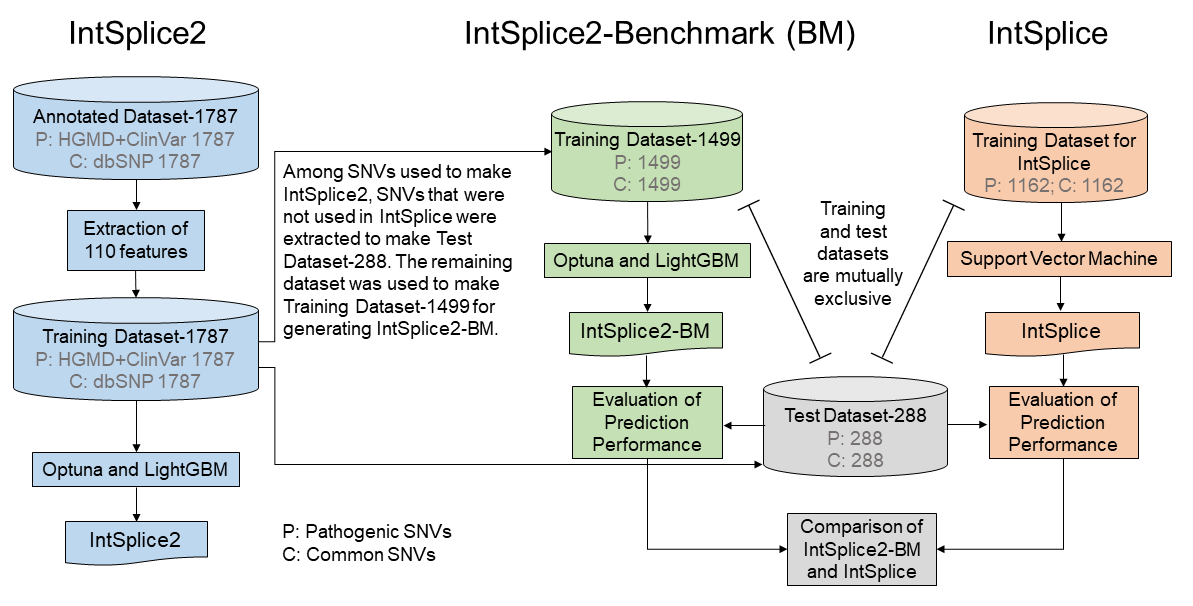

Supplement: Supplementary Figure 1 — The major pipelines of our analysis. [file Image_1.TIF]
