# Supplementary material for: IntSplice2: Prediction of the Splicing Effects of Intronic Single-Nucleotide Variants Using LightGBM Modeling
Source: Front Genet. 2021 Jul 19;12:701076. doi: 10.3389/fgene.2021.701076 (PMC8326971; doi:10.3389/fgene.2021.701076)
Supplement: Supplementary file 2 [file Data_Sheet_1.docx]

**Supplementary Table S1**. List of 110 features used to make IntSplice2 and IntSplice2-BM models.

| Features | 3’/Ex/5’ | Position^a^ | Feature importance^b^ | Rank of feature importance^b^ |
| --- | --- | --- | --- | --- |
| *Best-BPS^c^* |  |  |  |  |
| Number of nucleotides between the best BPS to Int-3 | 3' | Int-50 toInt-3 | 531 | 7 |
| Number of G nucleotides between the best BPS to Int-3 | 3' | Int-50 to Int-3 | 148 | 26 |
| Position weight matrix of the best BPS | 3' | Int-50 to Int-3 | 58 | 56 |
| *PPT* |  |  |  |  |
| Maximum length of polypyrimidines without any intervening purine | 3' | Int-50 to Int-3 | 102 | 40 |
| Maximum length of polypyrimidines allowing insertion of a single purine | 3' | Int-50 to Int-3 | 60 | 55 |
| Maximum length of polypyrimidines allowing insertion of two purines | 3' | Int-50 to Int-3 | 45 | 64 |
| Maximum length of polypyrimidines allowing insertion of three purines | 3' | Int-50 to Int-3 | 53 | 57 |
| Maximum length of polypyrimidines allowing insertion of four purines | 3' | Int-50 to Int-3 | 30 | 77 |
| Maximum length of T nucleotides without any intervening purine | 3' | Int-50 to Int-3 | 131 | 32 |
| Maximum length of T nucleotides allowing insertion of a single purine | 3' | Int-50 to Int-3 | 154 | 22 |
| Maximum length of T nucleotides allowing insertion of two purines | 3' | Int-50 to Int-3 | 106 | 38 |
| Maximum length of T nucleotides allowing insertion of three purines | 3' | Int-50 to Int-3 | 77 | 47 |
| Maximum length of T nucleotides allowing insertion of four purines | 3' | Int-50 to Int-3 | 169 | 20 |
| *Best-BPS-PPT^d^* |  |  |  |  |
| Position weight matrix of the BPS | 3' | Int-50 to Int-3 | 97 | 41 |
| Branch point is A at the best BPS | 3' | Int-50 to Int-3 | 0 | 94 |
| Ratio of pyrimidines (C/T) in PPT | 3' | Int-50 to Int-3 | 173 | 19 |
| Ratio of T in PPT | 3' | Int-50 to Int-3 | 175 | 18 |
| Ratio of G in PPT | 3' | Int-50 to Int-3 | 82 | 44 |
| Length of PPT | 3' | Int-50 to Int-3 | 139 | 30 |
| *Individual nucleotides* |  |  |  |  |
| A at Int-3 | 3' | Int-3 | 2412 | 4 |
| C at Int-3 | 3' | Int-3 | 717 | 6 |
| G at Int-3 | 3' | Int-3 | 7529 | 3 |
| T at Int-3 | 3' | Int-3 | 284 | 10 |
| A at the first nucleotide of exon | Ex | Ex+1 | 66 | 53 |
| C at the first nucleotide of exon | Ex | Ex+1 | 241 | 12 |
| G at the first nucleotide of exon | Ex | Ex+1 | 75 | 50 |
| T at the first nucleotide of exon | Ex | Ex+1 | 75 | 49 |
| Presence of A or G at Int-7, Int-6, or Int-5 | 3' | Int-7 to Int-5 | 199 | 17 |
| Ratio of purines (A/G) at Int-20 to Int-8 | 3' | Int-20 to Int-8 | 29 | 79 |
| Number of G nucleotides at Int-12 to Int-3 | 3' | Int-12 to Int-3 | 146 | 27 |
| Number of GGG trinucleotides at Int-12 to Int-3 | 3' | Int-12 to Int-3 | 35 | 72 |
| *Other parameters* |  |  |  |  |
| SD-Score | Ex/5' | Ex-3 to Int+6 | 221 | 15 |
| Exon length | Ex | Ex | 150 | 25 |
| MaxEntScan::score3ss | 3'/Ex | Int-20 to Ex+3 | 18982 | 2 |
| MaxEntScan::score5ss | Ex/5' | Ex-3 to Int+6 | 23 | 84 |
| Shapiro Senapathy score at 3’ ss | 3’/Ex | Int-14 to Ex+1 | 1059 | 5 |
| Shapiro Senapathy score at 5’ ss | Ex/5’ | Ex-2 to Int+6 | 103 | 39 |
| Gain of GT dinucleotide | 3' | Int-50 to Int-3 | 433 | 8 |
| Gain of AG dinucleotide | 3' | Int-50 to Int-3 | 19439 | 1 |
| *SpliceAid2 scores of RNA-bindingprotein^e^* |  |  |  |  |
| Sum score of 9G8-binding site | 3’/Ex/5’ | Int-50 to Int+50 | 35 | 71 |
| Sum score of CUG-BP1-binding site | 3’/Ex/5’ | Int-50 to Int+50 | 82 | 43 |
| Sum score of DAZAP1-binding site | 3’/Ex/5’ | Int-50 to Int+50 | 52 | 58 |
| Sum score of ESRP1-binding site | 3’/Ex/5’ | Int-50 to Int+50 | 235 | 14 |
| Sum score of ESRP2-binding site | 3’/Ex/5’ | Int-50 to Int+50 | 0 | 94 |
| Sum score of ETR-3-binding site | 3’/Ex/5’ | Int-50 to Int+50 | 151 | 24 |
| Sum score of FMRP-binding site | 3’/Ex/5’ | Int-50 to Int+50 | 34 | 73 |
| Sum score of Fox1-binding site | 3’/Ex/5’ | Int-50 to Int+50 | 25 | 82 |
| Sum score of Fox2-binding site | 3’/Ex/5’ | Int-50 to Int+50 | 0 | 94 |
| Sum score of HTra2alpha-binding site | 3’/Ex/5 | Int-50 to Int+50 | 37 | 70 |
| Sum score of HTra2beta1-binding site | 3’/Ex/5’ | Int-50 to Int+50 | 123 | 33 |
| Sum score of HuB-binding site | 3’/Ex/5’ | Int-50 to Int+50 | 45 | 63 |
| Sum score of HuC-binding site | 3’/Ex/5’ | Int-50 to Int+50 | 37 | 69 |
| Sum score of HuD-binding site | 3’/Ex/5’ | Int-50 to Int+50 | 50 | 60 |
| Sum score of HuR-binding site | 3’/Ex/5’ | Int-50 to Int+50 | 75 | 48 |
| Sum score of KSRP-binding site | 3’/Ex/5’ | Int-50 to Int+50 | 111 | 37 |
| Sum score of MBNL1-binding site | 3’/Ex/5’ | Int-50 to Int+50 | 160 | 21 |
| Sum score of Nova1-binding site | 3’/Ex/5’ | Int-50 to Int+50 | 315 | 9 |
| Sum score of Nova2-binding site | 3’/Ex/5’ | Int-50 to Int+50 | 271 | 11 |
| Sum score of PSF-binding site | 3’/Ex/5’ | Int-50 to Int+50 | 47 | 62 |
| Sum score of QKI-binding site | 3’/Ex/5’ | Int-50 to Int+50 | 0 | 94 |
| Sum score of RBM25-binding site | 3’/Ex/5’ | Int-50 to Int+50 | 0 | 94 |
| Sum score of RBM4-binding site | 3’/Ex/5’ | Int-50 to Int+50 | 0 | 94 |
| Sum score of RBM5-binding site | 3’/Ex/5’ | Int-50 to Int+50 | 50 | 59 |
| Sum score of SAP155-binding site | 3’/Ex/5’ | Int-50 to Int+50 | 0 | 94 |
| Sum score of SC35-binding site | 3’/Ex/5’ | Int-50 to Int+50 | 142 | 28 |
| Sum score of SF1-binding site | 3’/Ex/5’ | Int-50 to Int+50 | 0 | 94 |
| Sum score of SF2/ASF-binding site | 3’/Ex/5’ | Int-50 to Int+50 | 113 | 35 |
| Sum score of SLM1-binding site | 3’/Ex/5’ | Int-50 to Int+50 | 85 | 42 |
| Sum score of SLM2-binding site | 3’/Ex/5’ | Int-50 to Int+50 | 19 | 85 |
| Sum score of SRm160-binding site | 3’/Ex/5’ | Int-50 to Int+50 | 0 | 94 |
| Sum score of SRp20-binding site | 3’/Ex/5’ | Int-50 to Int+50 | 27 | 80 |
| Sum score of SRp30c-binding site | 3’/Ex/5’ | Int-50 to Int+50 | 39 | 68 |
| Sum score of SRp38-binding site | 3’/Ex/5’ | Int-50 to Int+50 | 1 | 92 |
| Sum score of SRp40-binding site | 3’/Ex/5’ | Int-50 to Int+50 | 30 | 76 |
| Sum score of SRp54-binding site | 3’/Ex/5’ | Int-50 to Int+50 | 0 | 94 |
| Sum score of SRp55-binding site | 3’/Ex/5’ | Int-50 to Int+50 | 154 | 23 |
| Sum score of SRp75-binding site | 3’/Ex/5’ | Int-50 to Int+50 | 13 | 88 |
| Sum score of Sam68-binding site | 3’/Ex/5’ | Int-50 to Int+50 | 43 | 65 |
| Sum score of TDP43-binding site | 3’/Ex/5’ | Int-50 to Int+50 | 5 | 90 |
| Sum score of TIA1-binding site | 3’/Ex/5’ | Int-50 to Int+50 | 30 | 75 |
| Sum score of TIAL1-binding site | 3’/Ex/5’ | Int-50 to Int+50 | 29 | 78 |
| Sum score of YB1-binding site | 3’/Ex/5’ | Int-50 to Int+50 | 111 | 36 |
| Sum score of ZRANB2-binding site | 3’/Ex/5’ | Int-50 to Int+50 | 41 | 66 |
| Sum score of hnRNP A0-binding site | 3’/Ex/5’ | Int-50 to Int+50 | 79 | 46 |
| Sum score of hnRNP A1-binding site | 3’/Ex/5’ | Int-50 to Int+50 | 12 | 89 |
| Sum score of hnRNP A2/B1-binding site | 3’/Ex/5’ | Int-50 to Int+50 | 116 | 34 |
| Sum score of hnRNP A3-binding site | 3’/Ex/5’ | Int-50 to Int+50 | 0 | 94 |
| Sum score of hnRNP C1-binding site | 3’/Ex/5’ | Int-50 to Int+50 | 71 | 52 |
| Sum score of hnRNP C2-binding site | 3’/Ex/5’ | Int-50 to Int+50 | 40 | 67 |
| Sum score of hnRNP C-binding site | 3’/Ex/5’ | Int-50 to Int+50 | 34 | 74 |
| Sum score of hnRNP D0-binding site | 3’/Ex/5’ | Int-50 to Int+50 | 49 | 61 |
| Sum score of hnRNP D-binding site | 3’/Ex/5’ | Int-50 to Int+50 | 23 | 83 |
| Sum score of hnRNP DL-binding site | 3’/Ex/5’ | Int-50 to Int+50 | 0 | 94 |
| Sum score of hnRNP E1-binding site | 3’/Ex/5’ | Int-50 to Int+50 | 16 | 87 |
| Sum score of hnRNP E2-binding site | 3’/Ex/5’ | Int-50 to Int+50 | 5 | 91 |
| Sum score of hnRNP F-binding site | 3’/Ex/5’ | Int-50 to Int+50 | 80 | 45 |
| Sum score of hnRNP G-binding site | 3’/Ex/5’ | Int-50 to Int+50 | 0 | 94 |
| Sum score of hnRNP H1-binding site | 3’/Ex/5’ | Int-50 to Int+50 | 221 | 16 |
| Sum score of hnRNP H2-binding site | 3’/Ex/5’ | Int-50 to Int+50 | 238 | 13 |
| Sum score of hnRNP H3-binding site | 3’/Ex/5’ | Int-50 to Int+50 | 73 | 51 |
| Sum score of hnRNP I (PTB)-binding site | 3’/Ex/5’ | Int-50 to Int+50 | 26 | 81 |
| Sum score of hnRNP J-binding site | 3’/Ex/5’ | Int-50 to Int+50 | 0 | 94 |
| Sum score of hnRNP K-binding site | 3’/Ex/5’ | Int-50 to Int+50 | 141 | 29 |
| Sum score of hnRNP L-binding site | 3’/Ex/5’ | Int-50 to Int+50 | 0 | 94 |
| Sum score of hnRNP LL-binding site | 3’/Ex/5’ | Int-50 to Int+50 | 0 | 94 |
| Sum score of hnRNP M-binding site | 3’/Ex/5’ | Int-50 to Int+50 | 0 | 94 |
| Sum score of hnRNP P (TLS)-binding site | 3’/Ex/5’ | Int-50 to Int+50 | 134 | 31 |
| Sum score of hnRNP Q-binding site | 3’/Ex/5’ | Int-50 to Int+50 | 17 | 86 |
| Sum score of hnRNP U-binding site | 3’/Ex/5’ | Int-50 to Int+50 | 65 | 54 |
| Sum score of nPTB-binding site | 3’/Ex/5’ | Int-50 to Int+50 | 0 | 93 |

^a^Postions to which the indicated parameter is applied. The number of nucleotides from the 5’ and 3’ ends of intron is indicated by Int+N and Int-N, respectively. The number of nucleotides from the 5’ and 3’ ends of exon is indicated by Ex+N and Ex-N, respectively.

^b^“Feature importance” is computed byLightGBM(Ke et al., 2017) after the hyperparameters are optimized by Optuna(Akiba et al., 2019), on Python version 3.8. The importance type is “gain” whose result contains total gains of splits which use the feature.

^c^Best-BPS, BPS with the highest PWM (position weight matrix) according to our report (Gao et al., 2008).

^d^Best-BPS-PPT, the best pair of BPS and PPT according to the following algorithm. First, ‘nYnAn’ motif is looked for with an invariant ‘A’ at Int-50:Int-3 and set to be BPS*_i_* (*i* Í N). Second, the ratio of T/C’s at positions +4 to +24 from the invariant ‘A’ of BPS*_i_* is calculated, while BPS*_i_* downstream of Int-9 is excluded because the length of putative PPT*_i_* becomes less than 7 nucleotides. This gives rise to multiple candidate BPS*_i_*-PPT*_i_* pairs. The sum of the PWM of BPS*_i_* and the T/C ratio in PPT*_i_* is then calculated and a pair with the best sum score is selected.

^e^The exact motif for an RNA-binding protein is searched for at Int-50:Ex:Int+50 and scored according to SpliceAid2 (Piva et al., 2012). The sum of SpliceAid2 scores is used as a parameter for each RNA-binding protein.

Methods to calculate individual parameters are available upon request.

**References**

Akiba, T., Sano, S., Yanase, T., Ohta, T., and Koyama, M. (2019). Optuna: A Next-generation Hyperparameter Optimization Framework. *arXiv* 1907.10902.

Gao, K., Masuda, A., Matsuura, T., and Ohno, K. (2008). Human branch point consensus sequence is yUnAy. *Nucleic Acids Res* 36**,** 2257-2267.

Ke, G., Meng, Q., Finley, T., Wang, T., Chen, W., Ma, W., Ye, Q., and Liu, T.Y. (2017). LightGBM: A Highly Efficient Gradient Boosting Decision Tree. *Adv. Neural Inf. Process Syst.*

Piva, F., Giulietti, M., Burini, A.B., and Principato, G. (2012). SpliceAid 2: a database of human splicing factors expression data and RNA target motifs. *Hum Mutat* 33**,** 81-85.

**Supplementary Table S2**. List of the optimized hyperparametersto make IntSplice2 model.

| Hyperparameters | Search space | The best value | Brief^a^ |
| --- | --- | --- | --- |
| lambda_l1 | 1e-8 to 10.0 | 6.409048325577845 | L1 regularization |
| lambda_l2 | 1e-8 to 10.0 | 0.12347125178592855 | L2 regularization |
| min_gain_to_split | 1e-8 to 10.0 | 2.1084284061817754e-07 | the minimal gain to perform split |
| num_leaves | 70 to 80 | 74 | max number of leaves in one tree |
| feature_fraction | 0.4 to 1.0 | 0.9162114923666314 | randomly select a subset of features on each iteration (tree) |
| bagging_fraction | 0.4 to 1.0 | 0.9972580371872813 | like feature_fraction, but this will randomly select part of data without resampling |
| bagging_freq | 1 to 10 | 7 | frequency for bagging |
| path_smooth | 1 to 10 | 9 | controls smoothing applied to tree nodes |
| min_data_in_leaf | 2 to 50 | 4 | minimal number of data in one leaf |
| min_sum_hessian_in_leaf | 1e-8 to 1.0 | 1.1298723965553308e-05 | minimal sum hessian in one leaf |
| max_depth | 3 to 7 | 7 | limit the max depth for tree model |
| max_bin | 50 to 255 | 232 | max number of bins that feature values will be bucketed in |
| learning_rate | 1e-3 to 1e-1 | 0.013739287004619802 | shrinkage rate |

Definitions of hyperparameters can be found in descriptions forLightGBM (https://lightgbm.readthedocs.io/en/latest/Parameters.html).

**Supplementary Table S3**. Area under the receiver operating characteristic curve (AUROC), area under the precision-recall curve (AUPR), and seven statistical measures of 5-fold cross-validation of IntSplice2 models trained using 1,787 pathogenic SNVs and 1,787 common SNVs with either 0.01 ≤ MAF < 0.50 or 0.01 ≤ MAF < 0.99.

| Common SNVs | AUROC | AUPR | Accuracy | Precision | Recall/Sensitivity | Specificity | F1 score | NPV | MCC |
| --- | --- | --- | --- | --- | --- | --- | --- | --- | --- |
| 0.01 ≤ MAF < 0.50 | 0.898 | 0.914 | 0.826 | 0.861 | 0.764 | 0.884 | 0.809 | 0.800 | 0.654 |
| 0.01 ≤ MAF < 0.99 | 0.885 | 0.896 | 0.820 | 0.828 | 0.792 | 0.846 | 0.809 | 0.813 | 0.639 |

**Supplementary Table S4. Comparison of statistical measures of aLightGBM-based model with those by fourother machine learning methods.**

**A. Fivemachine learning models were generated byTraining Dataset-1787 and were evaluated by 5-fold cross-validation.**

| Model | AUROC | AUPR | Accuracy | Precision | Recall/Sensitivity | Specificity | F1 score | NPV | MCC |
| --- | --- | --- | --- | --- | --- | --- | --- | --- | --- |
| LightGBM | 0.898 (1) | 0.914 (1) | 0.826 (1) | 0.861 (3) | 0.764 (2) | 0.884 (2) | 0.809 (1) | 0.800 (1) | 0.654 (1) |
| Random Forest (RF) | 0.883 (2) | 0.904 (2) | 0.817 (3) | 0.870 (1) | 0.743 (4) | 0.891 (1) | 0.801 (4) | 0.776 (4) | 0.640 (3) |
| Support Vector Machine (SVM) | 0.880 (3) | 0.897 (4) | 0.820 (2) | 0.864 (2) | 0.758 (3) | 0.882 (3) | 0.807 (2) | 0.784 (3) | 0.644 (2) |
| Extremely Randomized Trees (ERT) | 0.880 (3) | 0.900 (3) | 0.809 (4) | 0.861 (3) | 0.737 (5) | 0.882 (3) | 0.793 (5) | 0.770 (5) | 0.625 (4) |
| Multi-Layer Perceptron (MLP) | 0.870 (5) | 0.889 (5) | 0.807 (5) | 0.815 (5) | 0.791 (1) | 0.823 (5) | 0.803 (3) | 0.798 (2) | 0.613 (5) |

**B. Fivemachine learning models were generated by Training Dataset-1499and were evaluated by Test Dataset-288.**

| Model | AUROC | AUPR | Accuracy | Precision | Recall/Sensitivity | Specificity | F1 score | NPV | MCC |
| --- | --- | --- | --- | --- | --- | --- | --- | --- | --- |
| LightGBM | 0.890 (1) | 0.896 (2) | 0.826 (1) | 0.873 (1) | 0.764 (1) | 0.889 (2) | 0.815 (1) | 0.790 (1) | 0.658 (1) |
| Random Forest (RF) | 0.878 (2) | 0.897 (1) | 0.807 (2) | 0.870 (2) | 0.722 (3) | 0.892 (1) | 0.789 (2) | 0.763 (2) | 0.624 (2) |
| Support Vector Machine (SVM) | 0.856 (4) | 0.869 (4) | 0.781 (3) | 0.824 (4) | 0.715 (4) | 0.847 (4) | 0.766 (3) | 0.748 (4) | 0.567 (4) |
| Extremely Randomized Trees (ERT) | 0.867 (3) | 0.882 (3) | 0.780 (4) | 0.834 (3) | 0.698 (5) | 0.861 (3) | 0.760 (4) | 0.740 (5) | 0.567 (5) |
| Multi-Layer Perceptron (MLP) | 0.833 (5) | 0.850 (5) | 0.755 (5) | 0.753 (5) | 0.760 (2) | 0.750 (5) | 0.756 (5) | 0.768 (3) | 0.510 (3) |

The rank of each model in each statistical measure is indicated in parentheses.

The models are shown in descending order of average rank.

**Supplementary Table S5. Comparison of ninestatistical measures of IntSplice2-BM with those of three other toolsusing Test Dataset-288.**

| Tool | AUROC | AUPR | Accuracy | Precision | Recall/Sensitivity | Specificity | F1 score | NPV | MCC |
| --- | --- | --- | --- | --- | --- | --- | --- | --- | --- |
| S-CAP | 0.980 (1) | 0.985 (1) | 0.951 (1) | 0.939 (2) | 0.967 (1) | 0.935 (2) | 0.953 (1) | 0.965 (1) | 0.811 (1) |
| RegSNPs-intron | 0.837 (4) | 0.867 (4) | 0.850 (2) | 0.900 (3) | 0.793 (2) | 0.909 (3) | 0.843 (2) | 0.810 (2) | 0.690 (2) |
| TraP | 0.911 (2) | 0.925 (2) | 0.709 (4) | 0.992 (1) | 0.420 (4) | 0.997 (1) | 0.590 (4) | 0.633 (4) | 0.574 (4) |
| IntSplice2-BM | 0.890 (3) | 0.896 (3) | 0.826 (3) | 0.873 (4) | 0.764 (3) | 0.889 (4) | 0.815 (3) | 0.790 (3) | 0.658 (3) |

TraP: The thresholdfor possibly damaging classification was set to the default value (0.459).

S-CAP: The threshold of a model for the 3’ss intronicregion was set to high sensitivity (0.006).

RegSNPs-intron: Possibly damaging (PD) was regarded as damaging (D).

The rank of each model in each statistical measure is indicated in parentheses.

The models are shown in descending order of average rank.
